# An overview of gastrointestinal diseases in patients with COVID-19: A narrative review

**DOI:** 10.1097/MD.0000000000030297

**Published:** 2022-09-09

**Authors:** Cheng-Yao Lin, Shih-Bin Su, Kow-Tong Chen

**Affiliations:** a Division of Hematology-Oncology, Department of Internal Medicine, Chi-Mei Medical Center, Liouying, Taiwan; b Department of Senior Welfare and Services, Southern Taiwan University of Science and Technology, Tainan, Taiwan; c Department of Environmental and Occupational Health, National Cheng Kung University, Tainan, Taiwan; d Department of Occupational Medicine, Chi-Mei Medical Center, Tainan, Taiwan; e Department of Occupational Medicine, Tainan Municipal Hospital, Tainan, Taiwan; f Department of Public Health, College of Medicine, National Cheng Kung University, Tainan, Taiwan.

**Keywords:** COVID-19, epidemiology, gastrointestinal symptoms, pathophysiology

## Abstract

Coronavirus disease-2019 (COVID-19), caused by severe acute respiratory syndrome-coronavirus-2 (SARS-CoV-2), has emerged as a global health concern. This study aimed to review the epidemiology and pathophysiology of COVID-19 and provide evidence for the implementation of control measures.

We utilized several online databases, including MEDLINE (National Library of Medicine, Bethesda, Maryland, USA), PubMed, EMBASE, Web of Science, and Google Scholar, to collect relevant published papers using a combination of the following keywords: “COVID-19,” “SARS-CoV-2,” “novel coronavirus,” “epidemiology,” and “pathophysiology.” The Preferred Reporting Items for Systematic Reviews and Meta-Analyses (PRISMA) guidelines were used in this study.

Globally, approximately 3–46% of patients with SARS-CoV-2 infection experience gastrointestinal symptoms. The clinical spectrum of COVID-19 is wide, ranging from mild to severe, and even fatal. COVID-19 was initially reported as a respiratory tract disease; however, gastrointestinal symptoms have only recently been reported. COVID-19 Patients with gastrointestinal symptoms may have more severe clinical manifestations and poor prognosis.

This study highlights the need to better understand the mechanisms involved in the development of gastrointestinal symptoms in patients with COVID-19 to prevent the further spread of this pathogen.

## 1. Introduction

Coronaviruses (CoVs), the largest group of viruses within the order *Nidovirales*, comprise the *Coronaviridae, Aeteiciridae, Roniviridae*, and *Mesoniviridae*.^[1]^ CoVs are large, enveloped, single-stranded zoonotic RNA viruses.^[1]^ They can infect different animal species and cause serious diseases.^[2,3]^ In humans, CoVs mostly cause respiratory and gastrointestinal (GI) symptoms, ranging from the common cold to more severe diseases, such as pneumonia, severe acute respiratory distress syndrome (ARDS), and fatal illnesses.^[4–8]^ Human coronaviruses (HCoVs) have also been associated with exacerbations of chronic obstructive pulmonary disease,^[9]^ cystic fibrosis^[10]^ and asthma.^[11,12]^

The *Coronaviridae* family is further subdivided into 4 genera: α-, β-, γ-, and δ CoVs.^[8,13,14]^ Four strains of CoVs, HCoV2-229E, -HKU1, -NL63, and -OC43, have been found to circulate among humans.^[15,16]^ CoVs can also spread from animals to humans. They are characterized by rapid mutations and recombination, leading to the development of novel CoVs. Before 2002, coronaviruses were recognized as pathogens in animals and as the etiology of the common cold in humans. Between 2002 and 2003, a novel CoV emerged, causing severe acute respiratory syndrome (SARS).^[17–20]^ In addition to the SARS-CoV outbreak that occurred in 2002, Middle East respiratory syndrome coronavirus (MERS-CoV) emerged in Saudi Arabia in 2012.^[21,22]^ Severe acute respiratory syndrome-coronavirus-2 (SARS-CoV-2), which emerged in 2019, is the third novel coronavirus to infect human subjects.^[23]^ SARS-CoV-2 has 2 strains, bat-SL-CoVZC45 and bat-CoVZxC21, which share 88% genetic identity.^[24]^ The genetic sequence of SARS-CoV-2 has approximately 79% similarity to SARS-CoV-1 and 50% sequence similarity to MERS-CoV.^[23,25,26]^ Cross-species jumps from animals to humans with altered tropism are less likely to be due to genetic alterations. Environmental factors, frequency of human-animal contact, and globalization may influence the risk of cross-species infection.^[27]^

The epidemic of SARS-CoV-2 was first reported in Wuhan City, Hubei Province, China, on December 12, 2019. A local seafood and animal market was identified as a potential source of this outbreak.^[28]^ However, the main activators and transmission routes of this outbreak remain unclear.^[23]^ Following the epidemic occurrence of SARS-CoV-2 in China and its emergence as an international threat, the term “2019 coronavirus infection disease (COVID-19)” was announced on February 11, 2020, by the World Health Organization as the name of the clinical disease caused by SARS-CoV-2.^[29]^ COVID-19 has rapidly spread worldwide to become a global pandemic affecting over 79.2 million confirmed cases, and over 1.7 million deaths have occurred as of December 27, 2020.^[30]^

Typically, the primary symptoms of patients with COVID-19 are respiratory illnesses, including cough, dyspnea, and shortness of breath, although some patients suffer from GI symptoms, such as diarrhea, nausea/vomiting, and abdominal pain.^[31,32]^ In the United States, the first case of COVID-19 presented with a 2-day history of nausea and vomiting at the time of hospitalization,^[33]^ followed by diarrhea and abdominal pain on the second day of admission. SARS-CoV-2 RNA was detected in the feces on day 7 of the illness. In China, digestive symptoms were reported in COVID-19 patients during the initial outbreak.^[34,35]^ Additionally, a high proportion (up to 50%) of patients were found to have viral RNA present in their stool, even after viral RNA had been cleared from their respiratory tract.^[36,37]^ These results imply that SARS-CoV-2 actively infects and replicates in the gastrointestinal tract. Therefore, these findings have important implications for proper disease management, the potential fecal-oral route of transmission, and effective infection control.

## 2. Methods

In this study, we utilized several online databases, including MEDLINE (National Library of Medicine, Bethesda, Maryland, USA), PubMed, EMBASE, Web of Science, and Google Scholar, to collect relevant published papers using a combination of the following keywords: “COVID-19,” “SARS-CoV-2,” “novel coronavirus,” “epidemiology,” and “pathophysiology.” The review process followed the preferred reporting items for systematic reviews and meta-analyses (PRISMA) guidelines. We screened all the reference lists of relevant studies to identify any missing publications. The inclusion criteria for this review were observational studies reporting clinical symptoms at presentation in patients with COVID-19 (determined by nasopharyngeal swabs that were positive for SARS-CoV-2 via PCR) to estimate the prevalence of GI symptoms when present and observational studies providing data regarding RNA detection or the isolation of SARS-CoV-2 in stool samples of patients with COVID-19. Relevant original studies were quality-assessed by one of the investigators using a checklist developed by Hoogendoorn et al^[38]^ to evaluate observational studies. We defined high quality as a score of > 50% on the internal validity scale of the checklist. The articles reviewed in this report are limited to those published before January 2021. Articles that were not published in English, manuscripts without an abstract, or opinion articles were excluded. The literature review was conducted in February 2021.

As this review methodology aimed to synthesize information from available publications, ethical approval was not required.

## 3. Results and Discussions

**Figure 1.**
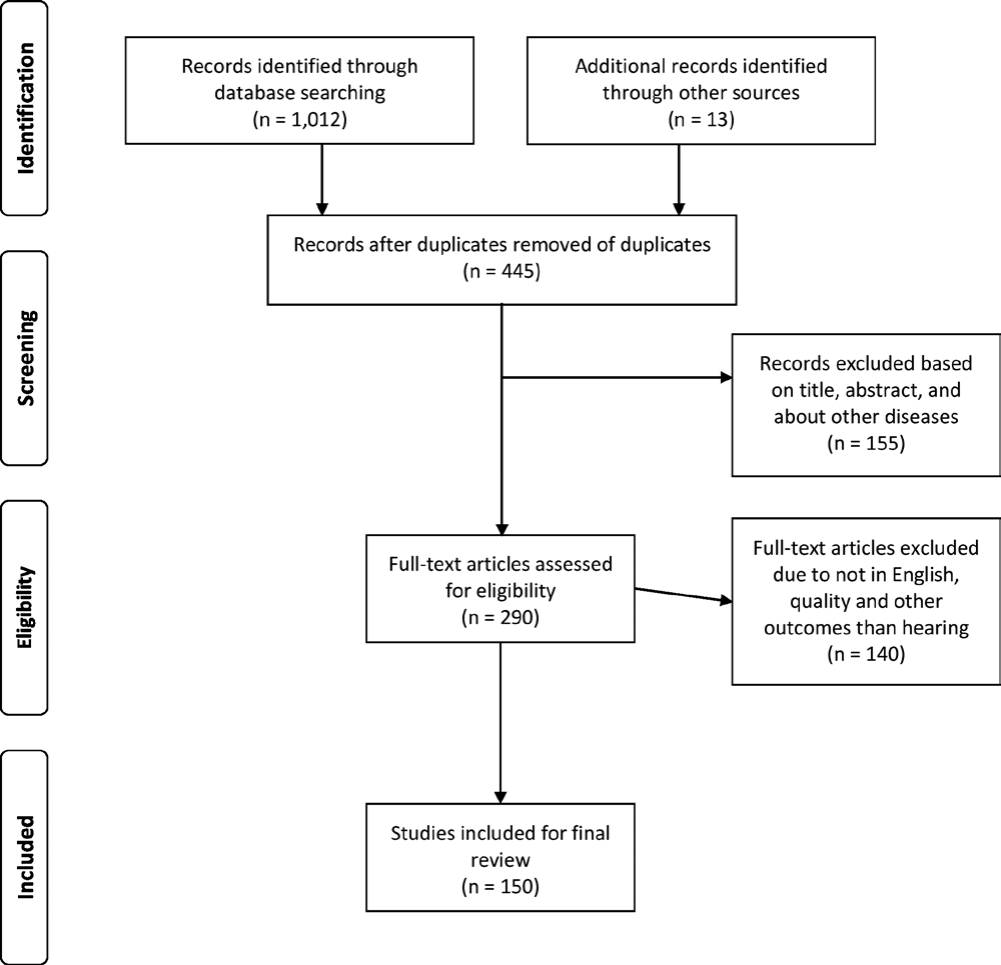
Flow diagram for the literature research in the study.

In this study, 1025 studies were collected from the aforementioned sources. All titles and abstracts from the literature search were independently assessed by 2 coauthors against the inclusion criteria for possible relevance. Discrepancies were resolved through consensus. All potentially relevant studies were read and evaluated by the authors. Finally, 150 studies were included in this review (Fig. 1). After the articles were selected, we collected all potential information related to epidemiology and pathophysiology, and classified the information accordingly.

## 4. Epidemiology

In the early stages of the SARS-CoV-2 outbreak, person-to-person transmission was suggested as the main route of transmission.^[23,39–41]^ COVID-19 is asymptomatic in some subjects, and in others, it can cause symptoms ranging from mild (>90%) to acute severe respiratory distress (ARDS), pneumonia, and fatality (approximately 1.6%).^[39–46]^ The reproductive number (R_0_) for SARS-CoV-2 was estimated at 2.7.^[41]^

Among patients hospitalized with SARS-CoV-2 infection, more than 70% are aged > 50 years, <5% are younger than 18 years, and the median age of hospitalized patients varies between 47 years (interquartile range [IQR]: 35–58) and 73 years (IQR: 58–82) in different reports.^[42–46]^ This result indicated that middle-aged and elderly individuals were the main people affected by the novel coronavirus.

Compared with adult COVID-19 patients, children with upper respiratory tract involvement have milder symptoms.^[43,45]^ However, the reason is still not clear why children patients with SARS-CoV-2 infection are having milder symptoms compared to the adult patients. This may be because children have lower immune responses to SARS-CoV-2 infection, partial immunity from other viral exposures, and lower rates of exposure to SARS-CoV-2. Although most children with COVID-19 are mild, a small portion (<7%) of children hospitalized with SARS-CoV-2 infection develop severe disease, requiring mechanical ventilation.^[47]^

A rare multiorgan inflammatory syndrome has also been described in children with COVID-19 in Europe.^[48,49]^ Multi-organ inflammatory syndrome, similar to Kawasaki disease in children, affects approximately 2 in 100,000 person-years, and there is a strong association between an outbreak of Kawasaki-like disease and the SARS-CoV-2 epidemic.^[48,49]^

Hospitalized patients are predominantly male, and the ratio of males to females is approximately 3:2,^[42,45,46]^ posing the question of why males are more susceptible to infection with SARS-CoV-19 than females. The angiotensin-converting enzyme 2 (ACE2) protein has been proven to be a cell receptor of SARS-CoV-1 and SARS-CoV-2 for mediating entry into host cells.^[50]^ ACE2 is an X-linked gene with a sex-specific expression profile.^[51]^ Males also have a higher rate of smoking than females, and smoking is related to higher expression of ACE2; therefore, it might also be another factor causing more severe COVID-19 cases in males than in females.^[52,53]^ However, whether ACE2 is linked to the clinical manifestations in patients with SARS-CoV-2 infection requires further investigation.

The mean incubation period for COVID-19 was approximately 5 days (IQR: 2–7 days).^[44,45]^ A high proportion (>90%) of individuals develop symptoms within 11.5 days of infection.^[44,45]^

COVID-19 presents with several clinical manifestations. In a study of 1099 patients with COVID-19 who had been hospitalized at 552 sites as of January 29, 2020,^[45]^ the most common symptom of COVID-19 was fever, followed by cough, nausea/vomiting, and diarrhea. Approximately 80% of patients present with mild symptoms, while 20% have severe disease; approximately 5% of patients present with severe symptoms, such as respiratory distress, sepsis, or multisystem dysfunction.^[45]^ However, some studies have reported that the most common symptoms at the time of hospitalization are fever, dry cough, shortness of breath, fatigue, nausea/vomiting, diarrhea, and myalgia.^[54,55]^ Recently, asymptomatic infections have been reported.^[56]^ It should be noted that some individuals with hidden symptoms or asymptomatic individuals are potentially infected without being aware of this, and they can infect multiple people.

It has also been reported that the most common comorbidity associated with more severe symptoms is hypertension, followed by diabetes, cardiovascular disease, chronic respiratory, hepatic, kidney diseases, and malignancy.^[43,45,54,55]^

In the United States, the first diagnosed case of COVID-19 presented with a 2-day history of nausea, vomiting, diarrhea, and abdominal pain upon hospitalization. SARS-CoV-2 RNA was detected in the feces on day 7 of the illness.^[33]^ This implies that SARS-CoV-2 can actively infect and replicate in the gastrointestinal tract. In China, digestive symptoms were reported in COVID-19 patients during the initial outbreak.^[34,35,57,58]^ This implies that SARS-CoV-2 can actively infect and replicate in the gastrointestinal tract.

The prevalence of GI symptoms reported in different countries is summarized in Table 1. The prevalence of GI symptoms in COVID-19 patients varies from country to country, from 3% in China to 45.8% in South Korea (Table 1).^[32,45,47,55,58–86]^ The most common GI symptom was diarrhea in both children and adults. A higher proportion (24.8%) of children exhibited vomiting than adults (16.7%).^[87]^ Furthermore, other GI symptoms noted were anorexia, vomiting, nausea, abdominal pain, and gastrointestinal bleeding (Table 1).

**Table 1:** A summary of the prevalence of gastrointestinal syndromes among patients with COVID-19 infection by year/month reported by country, 2020.

Year/month of reported	Countries/ regions	Population/sex/age	Prevalence (GI syndromes)	Reference
2020/2	China	Hospitalized patients (N = 41; M: 30, F: 11) Median age: 49.0 years (range: 41.0 to 58.0)	Diarrhea (3%)	Huang C, et al^[55]^
2020/3	China	Hospitalized patients (N = 292; Male: 119, Female: 72) Median age: 56.0 years (18–86)	Diarrhea (9%) Nausea/vomiting (4%)	Zhou F, et al^[58]^
2020/3	China	Hospitalized patients (N = 1099; M: 639, F: 460) Mean age: 47.0 years (IQR: 35–58)	Diarrhea (3.8%) Nausea/vomiting (5.0%)	Guan WJ, et al^[45]^
2020/4	China	Hospitalized patients (N = 168; M: 86, F: 82) Mean age: 56.7 years (±15.1)	Diarrhea 26.2%) Nausea (10.7%)	Meng Y, et al^[59]^
2020/4	Singapore	Hospitalized patients (N = 18; M: 9, F: 9) Median age: 47 (range: 31–73)	Diarrhea (17%)	Young BE, et al^[60]^
2020/4	Italy	Hospitalized patients (N = 44; M: 28, F: 16) Median age: 67.5 years (Range: 10–94)	Diarrhea (6.8%)	Colaneri M, et al^[61]^
2020/5	China	Hospitalized patients (N = 204; M: 107, Female: 97) Mean age: 52.9 years(±16)	Diarrhea (34%) Vomiting (3.9%) Abdomen pain (1.9%)	Pan L, et al^[34]^
2020/5	US	Hospitalized patients (N = 16; M: 12, F: 4) Median age: 67 years (range: 38–95)	Diarrhea (6%) Nausea/vomiting (13%)	Aggarwal S, et al^[62]^
2020/6	China	Hospitalized patients (N = 651; male: 331, female: 320) Mean age: 45.14 (±14.19)	Diarrhea (8.14%)	Jian X, et al^[32]^
2020/6	China	Hospitalized patients (N = 254, M: 115, F:139) Mean age: 50.6 years (range: 15–87)	Diarrhea (18.1%) Nausea (8.3%) Vomiting (5.9%)	Zhou Z, et al^[63]^
2020/6	China	Hospitalized patients (N = 206; M: 91, F: 115) Median age: 62.5 years (Range: 27–92)	Diarrhea (32.5%) Vomiting (11.7%)	Han C, et al^[64]^
2020/6	China	Multicenter Hospitalized patients (N = 1590; M: 57.3%) Mean age: 48.9 years (±16.3)	Diarrhea (4.2%) Nausea/vomiting (15.8%)	Liang WH, et al^[65]^
2020/6	China	Hospitalized patients (N = 95; male: 47.4%) Mean age: 45.3 years (±18.3)	Diarrhea (24.2%) Anorexia (17.9%) Nausea (17.9%)	Lin L, et al^[66]^
2020/6	US	Hospitalized patients (N = 150; male: 83, female: 67) Mean age: 57.6 years (±17.2) (with GI symptoms); 63.3 (±14.6) (without GI symptoms)	Diarrhea (14.7%) Nausea/vomiting (10.7%) Abdomen pain (2%)	Ramachandran P, et al^[67]^
2020/7	China	Hospitalized patients (N = 276; male: 155, female: 122) Median age: 51.0 years (45.0 to 58.0)	Diarrhea (2.2) Nausea/vomiting (8.3)	Wei Y, et al^[68]^
2020/7	Italy	Hospitalized patients (N = 34; M: 22, F: 12) Median age: 71 years (IQR: 59–81)	Diarrhea (2.9%) Nausea (2.9%) Abdomen pain (2.9%)	Papa A, et al^[69]^
2020/7	France	Hospitalized patients from 23 general pediatric hospitals (N = 192) Median age: 1 year (range: 0.125 to 11)	Diarrhea (16.7%) Vomiting (9.9%)	Gaborieau L, et al^[70]^
2020/7	Thailand	Hospitalized patients (N = 11; M: 6, F: 5) Median age: 61 years (rang: 28–74)	Diarrhea (18%) Vomiting (27%)	Pongpirul WA, et al^[71]^
2020/8	US	Hospitalized patients (N = 116; M: 53.4%) Median age: 50years (IQR, 35–67)	Diarrhea (12.0%) Nausea/vomiting (12.0%)	Cholankeril G, et al^[72]^
2020/8	US	Multicenter hospitalized patients (N = 318; M: 54.7%, F: 45.3%) Mean age: 63.4 years (±16.6)	Diarrhea (33.7%) Nausea (26.4%) Vomiting (15.4%) Abdomen pain (14.5%)	Redd WD, et al^[73]^
2020/9	China	Hospitalized patients (N = 465, M: 243, F: 222) Median age: 45 years (5–88)	Diarrhea (7.74) Nausea/vomiting (4.73)	Lian J, et al^[74]^
2020/9	European countries	Hospitalized patients (N = 582; M: 311, F: 271) Median age: 5.0 years (IQR: 0.5 to 12.0)	GI symptoms (22%)	Gotzinger F, et al^[47]^
2020/9	Chile	Hospitalized patients (N = 1155; M: 50%) Median age: 43.6 years (range: 23–83)	Diarrhea (7.3%) Abdomen pain (3.7%)	Diaz LA, et al^[75]^
2020/9	Japan (Diamond Princes cruise ship)	Hospitalizedpatients (N = 104; M: 54, F: 50) Median age: 68 years (range: 25–93)	Diarrhea (8.7%)	Tabata S, et al^[76]^
2020/9	Malaysia	Hospitalized patients (N = 247; Male: 172, Female: 75) Median age: 28 years (range: 18–35)	Diarrhea (9.7%) Nausea/vomiting (2.8%)	Soh TV, et al^[77]^
2020/9	South Korea	Hospitalized patients (N = 694; M: 212, F: 482) Mean age: 52.10 years (±18.29)	Diarrhea (23.9%)	Lee JY, et al^[78]^
2020/10	Brazil	Hospitalized patients (N = 400, M: 225, F: 175) Mean age: 56.40 (16.07)	Diarrhea (17.25%) Nausea (13.75%) Vomiting (7.5%) Anorexia (11.5%) Abdomen pain (6.0%)	Moura DTH, et al^[79]^
2020/10	Thailand	Hospitalized patients (N = 193; M: 113, F: 80) Median age: 37.0 years (IQR: 29.0 to 53.0)	Diarrhea (7.8%) Nausea/vomiting (2.6%)	Pongpirul WA, et al^[80]^
2020/10	Qatar	Hospitalized patients (N = 1409; M: 1167, F: 242) Median age: 35 years (IQR: 28–43)	Diarrhea (3.9%) Nausea/vomiting (3.8%)	Omrani A, et al^[81]^
2020/10	Turkey	Hospitalized children patients (N = 105; M: 51.4%) Mean age: 108.64 months(±65.61)	Diarrhea (4.8%) Vomiting (2.9%)	Yilmaz K, et al^[82]^
2020/11	South Korea	Hospitalized patients (N = 118; M: 52, F:66) Mean age: 61.0 years (50.0 to 70.0)	Diarrhea (45.8%)	Kang MK, et al^[83]^
2020/11	France	Hospitalized patients (N = 263; Male: 155, Female: 108) Median age: 65 years (range: 54–76)	Diarrhea (35.5%)	Jourdes A, et al^[84]^
2020/11	South Korea	Hospitalized patients (N = 7383; M: 39.8%, F: 60.2%) Mean age (44.0 years (±19.6)	Diarrhea (7.0%) Nausea (3.5%)	Shim E, et al^[85]^

In addition, a previous study indicated that SARS-CoV-2 nucleic acid was discovered in patients with COVID-19 feces^[33]^ and some patients with GI symptoms might develop severe symptoms. Investigating the epidemiological, clinical, and virological features of COVID-19 patients with GI symptoms is important for disease control and prevention. Previous studies revealed that the duration of GI symptoms among COVID-19 patients lasted from to 1-14 days (mean duration of 5.4 ± 3.1 days);^[57,64]^ COVID-19 patients with GI symptoms had a longer time from onset to hospitalization than those without GI symptoms (9.0 vs. 7.3 days).^[57]^ Other studies indicated that COVID-19 patients with GI symptoms had significantly higher rates of fever, fatigue, shortness of breath, and headache than patients with respiratory symptoms only, and COVID-19 patients with GI symptoms had a higher risk of family clustering and a longer duration of viral clearance than those with respiratory symptoms only.^[32,57,64]^

Patients with severe SARS-CoV-2 infection were found to have a higher risk of GI symptoms compared to those with mild symptoms.^[88,89]^ Some previous studies have also revealed that patients with COVID-19 admitted to the intensive care unit (ICU) were more likely to have GI symptoms than those without care in the ICU.^[32,89]^

COVID-19 Patients can also present with nonclassical symptoms. Olfactory and/or gustatory disorders have been reported in 64–80% of COVID-19 patients.^[90,91]^ Approximately 3% of the patients may have symptoms of anosmia or ageusia.^[92]^

There are various complications of COVID-19, including impaired function of the heart, brain, lung, liver, kidney, and coagulation system, COVID-19 can also lead to myocarditis, cardiomyopathy, ventricular arrhythmias, and hemodynamic instability.^[93,94]^ It has been observed that up to 8% of severe patients with complications of acute cerebrovascular disease and encephalitis^[91,95]^ and 10 to 25% of hospitalized patients with COVID-19 have venous and arterial thromboembolic events.^[96,97]^ In the ICU, venous and arterial thromboembolic events may occur in up to 31 to 59% of COVID-19 patients.^[97,98]^

Taken together, the prevalence of GI symptoms in COVID-19 varies among reports. Although COVID-19 mostly causes respiratory illnesses, it can also cause various GI symptoms. GI symptoms are generally more likely in severe COVID-19 cases, indicating the importance of the impact of GI symptoms on COVID-19 the spread and prognosis.

## 5. Pathophysiology

### 5.1. Involved target of SARS-cov2

When SARS-CoV-2 first emerged, several important questions arose: Why does COVID-19 spread so quickly, what is the role of the pathogenesis of the virus, what is the transmission route, how can large-scale spread be prevented, and what is the clinical impact of live SARS-CoV-2 virus detected in the stool on fecal–oral spread and infectivity?

SARS-CoV-2 has been detected in various samples, including whole blood, serum, urine, and stool.^[99,100]^ The virus is likely to infect respiratory epithelial cells and spread via droplets from humans to humans, causing a series of respiratory symptoms.^[100,101]^ SARS-CoV-2 can be detected in the respiratory tract of patients with COVID-19 1–2 days before the onset of clinical symptoms, and for up to 2 weeks after symptom onset.^[102–104]^

The mechanism of cell entry of CoVs has been extensively investigated. CoVs enter host cells through the binding of viral spike (S) proteins to cellular receptors and S protein priming by host cell proteases, which subsequently enter the endosomes and eventually fuse the viral and lysosomal membranes.^[105,106]^ The S-protein contains the S1 protein, which controls the binding receptor, and the S2 protein, which is responsible for membrane fusion.^[106]^ The SARS-CoV S protein comprises a receptor-binding domain (RBD) that specifically recognizes angiotensin-converting enzyme 2 (ACE2) as its receptor.^[107]^ Furthermore, the SARS-CoV spike protein requires proteolytic priming at the S1/S2 site by the type 2 transmembrane serine protease (TMPRSS2).^[50,108]^ This priming step is important for the fusion of the viral and cell membranes.^[50]^ Some other proteases (e.g., furin and cathepsin) may also play a role in SARS-CoV-2 priming and activating the entry of SARS-CoV-2.^[109]^

The RBD of the SARS-CoV-2 S glycoprotein is responsible for viral cell attachment to the ACE2 receptor and is a major determinant target of virus-neutralizing antibodies (NAbs).^[110]^ Protection against COVID-19 is largely mediated by an immune response directed against the SARS-CoV-2 S protein. Most vaccines that protect against COVID-19 have been developed on the basis of this concept. However, evolutionary biology is occurring globally, and a new mutated strain of SARS-CoV-2 (D614G) was reported in 2020.^[111]^ Alteration in the S protein may change its affinity for the ACE2 receptor. The mechanism of vaccine-induced immunity and variant susceptibility to neutralizing monoclonal antibodies requires further investigation.

SARS-CoV-2 and SARS-CoV-1 use the same receptor, ACE2, for entry into host cells.^[111]^ SARS-CoV-2 entry into host epithelial cells is dependent on the ability of viral spike (S) proteins to bind to ACE2.^[105]^ ACE2 is found on the apical membranes of the nasal, oral, nasopharyngeal, and oropharyngeal mucosal epithelium; alveolar epithelium; endothelial cells of blood vessels and the heart; renal tubules; and enterocytes in the small intestine.^[24,112]^ Early in the infection process, SARS-CoV-2, through the viral structural spike (S) protein, binds to the ACE2 receptor on nasal epithelial cells, bronchial epithelial cells, or lung cells.^[50]^ A study by Wang et al^[113]^ indicated that more than 80% of ACE2-expressing pulmonary cells are type II alveolar cells. This cell type may be a potential site of coronavirus invasion and replication. Rapid replication of SARS-CoV-2 in the lungs may trigger a cytokine storm reaction and respiratory disorders.^[55]^ In addition, TMPRSS2 in the host cell facilitates viral uptake by proteolytic cleavage at the S1/S2 and S2 sites of ACE2 and activates the fusion of SARS-CoV-2 and the host membrane.^[105]^ SARS-CoV-2 has a higher affinity (approximately 10–20 times) than SARS-CoV-1^[114]^ which may explain why SARS-CoV-2 has higher infectivity but less fatality than SARS-CoV-1.

Taken together, the expression of ACE2 is essential for the entry of SARS-CoV-2 into host cells. These disease outcomes might be due to changes in the functional activity of ACE2. In humans, ACE2 and TMPRESS2 play pivotal roles in the lung tissue and epithelial cells. This finding explains why the lungs appear to be the most vulnerable target organ for SARS-CoV-2 infection. In addition to the tissue of the respiratory tract, ACE2 is also found in many extrapulmonary organs, including the heart, kidney, and intestine.^[115]^

### 5.2. Involved target of SARS-cov2 in GI tract

Pathological manifestations of the intestinal tract due to SARS-CoV-2 infection have been identified through autopsy and biopsy. The intestinal autopsy of a COVID-19 patient showed that the patient presented with alternating segmental dilatation and stenosis of the small intestine.^[116]^

Previous studies have revealed that SARS-CoV-2 RNA was detected in 7–50% of stool specimens among hospitalized patients with COVID-19.^[33,117,118]^ These findings suggested that the virus can grow and survive in the digestive tract. Compared with subjects presenting solely with respiratory symptoms, COVID-19 patients with GI symptoms (diarrhea, nausea, and vomiting) presented for care later (11.6 ± 5.1 vs. 16.0 ± 7.7 days, *P* < .001), and their fecal test positivity was higher in those with GI symptoms (73%) than in those with only respiratory symptoms (14%).^[80]^ Notably, some COVID-19 patients still present positivity for nucleic acids in stool after pharyngeal swab, indicating a negative result.^[119,120]^ Compared with patients with positive respiratory samples, patients with positive fecal samples for SARS-CoV-2 RNA remained positive for SARS-CoV-2 RNA longer (27.7 ± 10.7 days vs. 16.7 ± 6.7 days) after first symptom onset.^[119]^ On average, SARS-CoV-2 shedding from feces can continue for up to 11 days after respiratory symptoms subside.^[121]^ This finding cannot be interpreted as the temporary gastrointestinal transit of swallowed saliva containing the virus. These findings imply that the GI tract may be involved in the SARS-CoV-2 infection.

Additionally, in 1 study, several examinations were conducted to further investigate the GI tract in patients with SARS-CoV-2 infection, such as GI endoscopy for patients who were diagnosed with positive findings of SARS-CoV-2 infection from stool examinations, and histopathologic and immunofluorescent staining for patients who had biopsy specimens obtained from the esophagus, gastric, duodenum, and colon tissues. In this study, SARS-CoV-2 RNA was identified by positive staining of the viral nucleocapsid protein in the gastric, duodenal, and colonic epithelium upon endoscopy and biopsy.^[117]^ These findings indicate that SARS-CoV-2 may infect the mucosal cells of the stomach and small and large intestines, replicate, and produce infectious virions. These findings suggest that the GI tract may be a favorable organ for viral growth, with a potential fecal-oral route of transmission.

One previous study revealed that it was a possible risk that endoscopists face exposure to potentially infectious virus specimens during endoscopy.^[122]^ This exposure may cause transmission of infectious agents. GI endoscopists come into close contact with the upper and lower GI tract contents, especially those through the nasal and oral cavities, and this may induce coughing and subsequent emission of droplets. These factors can increase the risk of infectivity among healthcare workers and contribute to viral infections.^[123]^

### 5.3. Potential theories of GI disorders in COVID-19

Through direct viral invasion, the gut epithelium may cause inflammation and damage.^[124]^ Once the virus is infected, the permeability of the GI wall to pathogens might change, and GI symptoms (e.g., diarrhea) will occur due to enterocyte malabsorption. This indicates that the GI tract may be vulnerable to SARS-CoV-2 infection.^[125]^ COVID-19 with GI symptoms may develop ACE2 dysfunction. A previous study indicated that ACE2 is a pivotal regulator of the renin-angiotensin-aldosterone system (RAAS) and influences inflammatory reactions and immune functions.^[126]^ It was found that ACE2 messenger RNA is highly expressed and the encoded protein heterodimers with the neutral amino acid transporter broad neutral amino acid transporter 1 (B^0^AT1) (SLC6A19) or the imino acid transporter sodium-dependent imino transporter 1 (SIT1) (SLC6A20) in the GI tract.^[91,127]^ They can provide potential binding sites for SARS-CoV-2 spike glycoproteins and subsequent infections. ACE2 with B^0^AT1 is suggested to provide the substrate amino acids to the transporter; however, further studies are needed to prove whether this correlation influences the function of ACE2 in mediating viral infection of the transporter.

It has been found that SARS-CoV-2 may affect the central nervous system.^[91]^ Regarding the theory of the gut-brain axis, it needs further investigate the role of the gut-brain axis in GI disorders during SARS-CoV-2 infection.

### 5.4. The possible role of the GI microbiota in SARS-cov-2 infection

**Figure 2.**
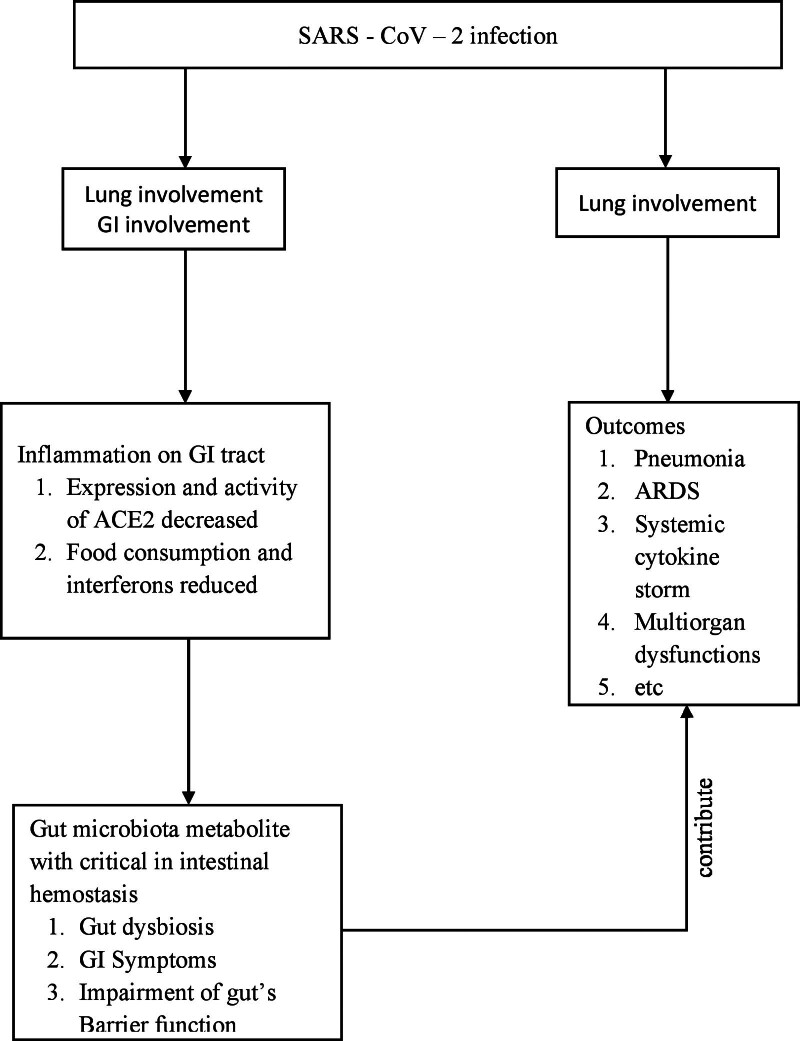
Possible role of gut microbiota in SARS-CoV-2 infection.^[128–145]^

Previous studies indicate that SARS-CoV-2 infection involving the GI tract includes the possibility of a systemic inflammatory response to the gut secondary to viremia, which may lead to an alteration of the gut microbiota. Paradoxically, dysbiosis of microbiota can worsen the prognosis of the disease (Fig. 2).^[128–145]^ The Gut microbiota plays a crucial role in regulating intestinal mucosal immunity and maintaining intestinal homeostasis in both healthy and diseased states.^[128]^ Short-chain fatty acids (SCFAs), bile acids, and the essential aromatic amino acid tryptophan are considered important metabolites involved in the interactions between gut microbiota and the host.^[128,129]^ SARS-CoV-2 uses the ACE2 receptor to enter the host, which is highly expressed in both the respiratory and GI tracts.^[130,131]^ ACE2 plays an important role in controlling intestinal inflammation and the gut microbial ecology.^[132]^ The commensal microbiota ecosystem in the gut is dynamic and can be regulated by invading viruses to facilitate stimulatory or suppressive responses.^[133]^ Microbial dysbiosis has been identified in patients with respiratory tract infections, which predispose them to secondary bacterial infections.^[134,135]^

Dysbiosis, the dysfunction of intestinal microbes, is associated with various human disorders. As recently studied in patients with sepsis and acute respiratory distress syndrome (ARDS), the gut-lung axis is bidirectional, and endotoxins and microbial metabolites and the lung microbiome may become enriched with gut-associated microbes.^[136]^ This, in turn, may affect the lung microbiota via the “gut-lung axis,” increasing the risk of developing acute respiratory distress syndrome.^[137]^ Gut-lung interactions have been described in patients with respiratory infections. For example, infection with the influenza A virus is associated with intestinal disorders and alterations in the gut microbiota.^[42]^ It has been suggested that a reduction in the production of short-chain fatty acids (SCFA) is associated with a decrease in the bactericidal activity of alveolar macrophages.^[138]^

Several studies have shown that respiratory viral infections are associated with changes in the intestinal microecology.^[134,139]^ Compared to healthy individuals, patients with COVID-19 have significant differences in fecal microbiota.^[133,140]^ The increase in opportunistic pathogens and the decrease in beneficial commensals, including *lactobacilli* and *bifidobacteria*, in the lung have been associated with systemic inflammation markers and the occurrence of sepsis.^[141,142]^

The microbiota in the GI tract is a prosperous and diverse ecosystem that is associated with many functions of the GI system, as well as the pathogenesis of the GI tract. In a previous study using bronchoalveolar lavage fluid, it was found that the microbiota in patients with SARS-CoV-2 infection was either dominated by pathogens or was related to increasing concentrations of oral and upper respiratory commensal bacteria.^[143]^ The Alterations in the microbiota ecosystem in the GI tract persisted even after clearance of SARS-CoV-2 and remission of respiratory symptoms.^[133,143]^ In addition, the morbidity of comorbidities associated with severe COVID-19 is related to changes in the relative enrichment of Bacteroidetes and Firmicutes.^[144]^ It has been shown that these microbiomes are an important risk factor for gut microbiota during the formation of the immune system.^[145]^ Accordingly, we propose that the intestinal microbiota is associated with susceptibility to SARS-CoV-2 infection and development of severe disease.^[133]^

Probiotics are live microorganisms that are advantageous for patient immunity when administered in adequate amounts. Probiotics are recommended for the prevention and treatment of GI infections and diseases.^[146]^ In recent years, probiotics have been increasingly recognized as useful tools for the prevention and control of respiratory tract infections.^[147]^ The previous study showed that the effects of probiotics are mediated through immune regulation, and that they help maintain the integrity of the junctions between enterocytes to prevent the entrance of SARS-CoV-2.^[148]^

Nutritional support and the application of prebiotics or probiotics have been recommended for patients with SARS-CoV-2-infected to adjust the balance of the intestinal microbiota and reduce the risk of secondary bacterial infections.^[148,149]^ Previous studies have shown that probiotics may shorten the duration of acute respiratory infections and reduce the rate of ventilator-associated pneumonia in patients on severe mechanical ventilation.^[150]^ However, more evidence is needed to support the use of probiotics for the prevention and treatment of patients with SARS-CoV-2 infection in the future.^[142]^

Taken together, these findings support the replication of infectious virions within the GI tract. In addition, SARS-CoV-2 RNA in stool can survive even after viral RNA in the respiratory tract is cleared. Accordingly, fecal-oral transmission is a potential source for the spread of SARS-CoV-2 throughout the population. It should be noted that the guidelines of care for COVID-19 patients leaving the hospital should include fecal viral examinations because of delayed elimination. Testing for viral RNA in feces using rRT-PCR should be used for adequate source and infection control.

## 6. Conclusions

SARS-CoV-2 uses both the respiratory and fecal-oral routes for efficient transmission. SARS-CoV-2 RNA can survive longer in stool specimens than in respiratory specimens, and this may serve as evidence for GI tract viral replication and subsequent shedding. Patients with COVID-19 and GI symptoms may have more severe disease and poorer outcomes. Therefore, COVID-19 patients with GI symptoms should be advised to practice proper hand hygiene and maintain social distancing to prevent and control SARS-CoV-2 infection. The testing of viral RNA in feces by rRT-PCR may be an alternative way to monitor infectious sources. A better understanding of the mechanisms associated with the development of GI symptoms is necessary to identify the most appropriate approach for COVID-19 prevention and treatment.

## Author contributions

Conceptualization: Kow-Tong Chen.

Data curation: Cheng-Yao Lin.

Formal analysis: Cheng-Yao Lin.

Methodology: Cheng-Yao Lin, Shih-Bin Su, and Kow-Tong Chen.

Supervision: Shih-Bin Su and Kow-Tong Chen.

Validation: Cheng-Yao Lin.

Writing – Original draft: Cheng-Yao Lin and Kow-Tong Chen.

Writing – Review and editing: Kow-Tong Chen.
